# Blood storage effect of G6PD on RBC quality

**DOI:** 10.1016/j.htct.2025.103733

**Published:** 2025-05-13

**Authors:** Andrew Evans Cobbinah, Benedict Sackey, Mina Ofosu, Herbert Ekoe Dankluvi, Stephen Opoku, Ampa Davis Frank

**Affiliations:** a Kwame Nkrumah University of Science and Technology, Kumasi, Ghana; b Kumasi Technical University, Kumasi, Ghana; c Blood Bank Department, Living Waters Hospital, Ejisu, Ghana

**Keywords:** G6PD, Oxidative stress, Hemolysis, Cellular membrane, Homeostasis

## Abstract

**Background:**

The most prevalent metabolic condition of red blood cells, glucose-6-phosphate dehydrogenase (G6PD) deficiency, affects around 35 million people globally. The highest prevalence is seen in tropical and subtropical areas of the eastern hemisphere, where it can affect up to 35 % of the population. G6PD deficiency, the most prevalent enzyme deficit, is not currently tested for in blood products. G6PD deficiency is a genetic factor that influences the quality of stored red blood cells impacting their ability to respond to oxidative stress. This hospital-based cross-sectional study aimed at assessing the prevalence of G6PD deficiency in donor blood and the impact of the enzyme deficiency on red cell indices during storage.

**Method:**

A total of 57 blood bags were screened for G6PD deficiency. Red cell indices and blood film comments were investigated on Day 0, Day 7 and Day 14 of storage.

**Results:**

Eight out of 57 (14 %) had the G6PD full defect and 86 % (49/57) had no defect. Over the course of 14 days storage, the hemoglobin and red blood cell count significantly decreased in G6PD-deficient blood units with a corresponding significant increase in mean corpuscular volume and red cell distribution width-standard deviation compared to baseline and normal G6PD activity. The blood film comment showed 85.7 % normocytic normochromic, 2.0 % microcytic hypochromic and 12.2 % macrocytic hyperchromic from G6PD-non-deficient donors whereas G6PD-deficient donors had 75 % normocytic normochromic with 12.5 % microcytic hypochromic and 12.5 % macrocytic hypochromic after 2 wk in storage.

**Conclusion:**

Red blood cell count and hemoglobin reduce significantly in G6PD-deficient donor units during storage with an associated increased mean corpuscular volume indicating progressive loss of the cellular membrane homeostatic mechanism that could potentially result in further hemolysis during long term storage.

## Introduction

One of the most common therapies for anemic hospitalized patients is red blood cell (RBC) transfusions.^1^ Patients with sickle cell disease and thalassemia, in particular, require chronic transfusions because of inherent RBC abnormalities linked to increased hemolysis and inefficient erythropoiesis. Accelerated clearance of transfused RBCs results in several side effects related to continuous RBC transfusion therapy, including iron overload, alloimmunization, and perhaps increased susceptibility to infection.^2^ As a consequence, numerous initiatives are made to supply the highest quality RBC products. The Food and Drug Administration (FDA) establishes acceptance criteria for RBC units at the end of their maximum permitted storage period (42 days), which are primarily based on an average 24-hour post-transfusion recovery (PTR) rate of at least 75 % (i.e., 75 % of the transfused RBCs should still be circulating 24 h after transfusion) and a <1 % rate of in vitro hemolysis.^3^ Additionally, the proportion of successful PTRs must have a one-sided, lower limit of the 95 % confidence interval of at least 70 %; in other words, there can be no more than two unsuccessful PTRs of 75 % in a cohort of 20 healthy volunteer blood donors.

PTRs are remarkably different between blood donors,^4^ with these variations being distinct and recurrence-free for each donor, indicating that some donors are strong iron storers and others are poor iron storers.^1^ Inter-donor metabolic heterogeneity was discovered by in vitro tests of preserved RBCs; this heterogeneity can affect the metabolic age of stored RBC units at least as much as their chronological age.^5^ Furthermore, as RBC storage quality is heriTable,^6^ genetic factors might be to blame for at least some of these variances.

The most prevalent human enzymopathy, glucose-6-phosphate dehydrogenase (G6PD) deficiency, is an X-linked illness that affects around 400 million people worldwide.^7^ The pentose phosphate pathway (PPP), which produces reduced nicotinamide adenine dinucleotide phosphate (NADPH), a cofactor that powers a number of antioxidant pathways in RBCs, also depends on G6PD as its rate-limiting enzyme.^8^ In fact, NADPH is necessary for glutathione reductase to recycle oxidized glutathione into its reduced form. The thioredoxin reductase system, biliverdin reductase B, and the ascorbate-tocopherol axis are just a few examples of the numerous NADPH-dependent antioxidant enzymes it supports.^9^ It also enhances catalase, glutathione peroxidase, peroxiredoxins, glutaredoxins, and the thioredoxin reductase system. The reduced ability of G6PD-deficient RBCs to produce NADPH,^1^ which can be brought on by drugs, infections, and nutrition, makes them more vulnerable to oxidative stress.^10^

In refrigerated storage, oxidative stress indicators increase,^11^^,^^12^ indicating that storage itself may contribute to oxidative stress. PTR also increases noticeably in mice and humans when RBCs are maintained under hypoxic conditions^13^ or in the presence of the antioxidant ascorbic acid,^14^ which reduces oxidative stress. RBCs do not appear to have evolved to withstand the oxidative damage brought on by cold storage however, they evolved defenses against oxidative stress as they age in vivo with some of these defenses being triggered during typical blood bank storage. Studies using stable isotope-labeled tracers, for instance, indicate that storage-induced oxidation of Cys152 of the glycolytic enzyme glyceraldehyde 3-phosphate dehydrogenase (GAPDH) results in a shift in the glucose metabolism toward the oxidative phase of the PPP; this phenomenon is attenuated or exacerbated by hypoxic or hyperoxic storage, respectively.^15^ G6PD-deficiency reduces NADPH generation in RBCs, which reduces their capacity to replenish the reduced form of glutathione and prevent the buildup of peroxidation/inflammatory products.^16^ G6PD is the most important enzyme in the oxidative phase of the PPP. In fact, blood units obtained from G6PD-deficient donors have altered glutathione homeostasis and antioxidant defenses.^17^

## Method

### Study design

This was a cross-sectional study to assess the prevalence of G6PD deficiency among blood donors. It also has a comparative study design to assess the impact of G6PD deficiency on stored RBCs as compared to non-G6PD-deficient stored RBCs.

### Ethical considerations

Ethical clearance was obtained from the Committee on Human Research Publications and Ethics (CHRPE) of the School of Medicine and Dentistry, Kwame Nkrumah University of Science and Technology before the inception of the study. The management of Living Waters Hospital also gave their approval for their facility to be used for this study. Moreover, consent was sought from blood donors who were assured of the highly confidential nature of this study.

### Sample collection

About 5 mL of blood was collected from each blood unit donated in the blood bank from patients who had passed the donor screening tests. These samples were used for the initial analysis. Subsequently after 7 and 14 days, additional samples were collected from the same blood bags that had been kept in a storage fridge.

The first set of samples were screened for G6PD deficiency using the methemoglobin reductase technique. Thin films were prepared, stained with Leishman stain and observed for general film comment on the red cell morphology. Furthermore, a complete blood count was performed on the samples to determine red cell hematological indices.

### Laboratory investigations

#### The procedure of the G6PD screening test

The methemoglobin technique of G6PD testing was done by arranging three test tubes in a test tube rack with the labels ‘Positive’, ‘Test’ and ‘Negative’. One mL each of a well-mixed blood sample from a CPD-A1 anti-coagulant blood storage bag was introduced into the three test tubes. Fifty µL of a mixture of sodium nitrite and glucose was dispensed into the tubes labelled ‘positive’ and ‘test’ and mixed and 50 µL of methylene blue was added to the tubes labelled ‘test’ and ‘negative’ and mixed.

The test tube setups were then corked and incubated in a water bath at 37 °C for 3 h. At the end of this time, the contents of the tubes were diluted with physiological saline solution and observed against a white background. The result was read as either full defect, partial defect or no defect.

#### Complete blood count

The blood sample collected from the blood bags into a plain test tube was swirled to evenly distribute blood cells.

Following standard protocols, the complete blood count of all samples was analyzed using a MINDRY BC-3000Plus 3PARTS Automated Hematology Analyser from the Kumasi Technical University Clinic laboratory.

The parameters of interest of the complete blood count analysis were the hemoglobin (Hb) concentration, RBC count, mean corpuscular volume (MCV), mean corpuscular Hb (MCH) and mean corpuscular Hb concentration (MCHC) since the study focuses on RBC indices.

#### Blood film comment

Thin blood films of each sample were prepared and stained with Leishman stain using the standard staining protocol, with Leishman stain being flooded on the smear for 1–2 mins and then diluted with buffered water at about twice the volume of the stain and allowed to stand for 15 mins. The slides were then washed and blotted for observation.

The stained slides were observed by a student and the blood picture was confirmed by an independent experienced hematologist at the facility. The observed morphological characteristics of the cells were then used to categorize the cells.

## Results

### Socio-demographic characteristics of study participants

A total of 57 male blood donors were recruited for this study. The mean age of the blood donors was 26.47 ± 3.723 years (range: 19–38 years). The majority of the blood donors were in the 21–25 (46.6 %) age group followed by 26–30 (36.2 %), whilst the smallest age group was that of 36–40 (1.8 %) years old. Of the various blood groups, 45.6 % were of the *O*^+^ blood group, followed by 24.6 %, 17.5 %, 5.3 %, 3.5 %, 1.8 % and 1.8 % of the *A*^+^, *B*^+^, AB^+^, *B*^−^, *A*^−^ and *O*^−^ blood groups, respectively.

**Figure 1:**
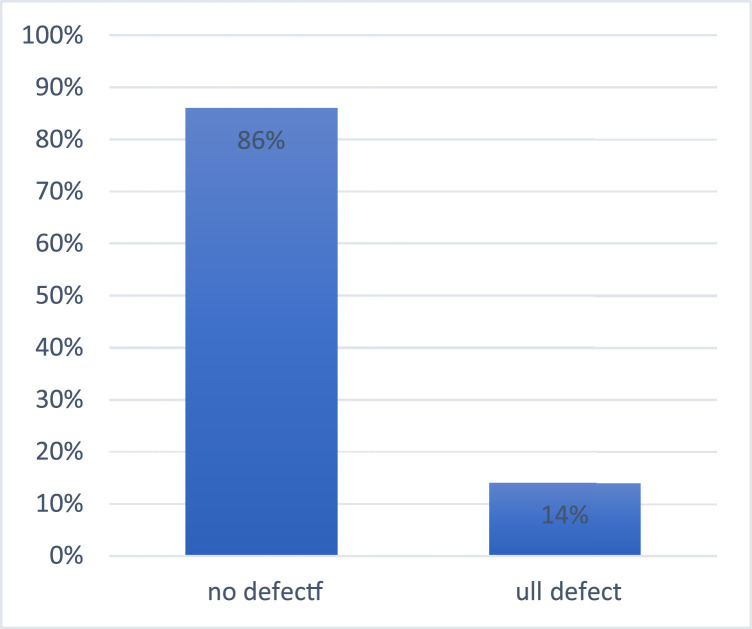
Prevalence of G6PD status among blood donors.

From the total of 57 blood donors recruited, 8 (14 %) had the full defect for G6PD enzyme activity whilst 49 (86 %) had no defect for G6PD activity. This gives a 14 % (8/57) prevalence of G6PD deficiency among blood donors of this study (Table 1 and Figure 1).

**Table 1:** Shows descriptive statistics of the blood donors in the study.

Variable	Frequency	Percentage (%)
Gender		
Male	57	100
Female	0	0
Total	57	100
Age group-years		
16–20	2	3.5
21–25	27	47.4
26–30	21	36.8
31–35	6	10.5
36–40	1	1.8
Total	57	100
Blood group		
A-	1	1.8
*A*+	14	24.6
AB+	3	5.3
B-	2	3.5
*B*+	10	17.5
O-	1	1.8
*O*+	26	45.6
Total	57	100
G6PD Status		
No defect	49	86
Full defect	8	14
Total	57	100

### General effect of storage on RBC indices of donor blood

At baseline, the mean Hb, RBC count, MCV, MCH, MCHC and red cell distribution width-standard deviation (RDW-SD) of the donor units were 13.00 ± 1.99 g/dL, 4.55 ± 0.62 × 10^12^/L, 82.57 ± 9.71 fL, 27.48 ± 4.36 pg, 33.10 ± 2.22 g/dL and 48.16 ± 3.5 fL, respectively (Table 2).

**Table 2:** Changes in red blood cell parameters of donor blood over a 7-day storage period.

Variable	Baseline	7 days of storage	*p*-value
Hb (g/dL)	13.00 ± 1.99	12.78 ± 2.26	0.023
RBC (×10^12^/L)	4.55 ± 0.62	4.50 ± 0.66	0.368
MCV (fL)	82.57 ± 9.71	83.43 ± 10.91	0.22
MCH (pg)	27.48 ± 4.36	27.21 ± 4.41	0.336
MCHC (g/dL)	33.10 ± 2.22	32.60 ± 1.61	0.08
RDW-SD (fL)	48.16 ± 3.5	50.24 ± 4.1	0.00

Hb: hemoglobin; RBC: Red blood cell; MCV: mean corpuscular volume; MCH: mean corpuscular hemoglobin; MCHC: mean corpuscular hemoglobin concentration; RDW-SD: red cell distribution width-standard deviation.

Comparing the hematological indices of the donor samples from the baseline to Day 7 in storage, the mean Hb decreased significantly (*p*-value = 0.023) from 13.00 ± 1.99 g/dL to 12.78 ± 2.26 g/dL while the RDW increased significantly (*p*-value = 0.00) from 48.16 ± 3.5 fL to 50.24 ± 4.1 fL. However, the RBC count (*p*-value = 0.368), MCV (*p*-value = 0.220), MCH (*p*-value = 0.336) and MCHC (*p*-value = 0.080) showed no significant changes (Table 2).

Comparing the data again from the baseline to day 14 in storage, the mean Hb and MCHC decreased significantly from 13.00 ± 1.99 g/dL to 12.87 ± 2.57 g/dL (*p*-value = 0.009) and 33.10 ± 2.22 g/dL to 30.90 ± 2.08 g/dL (*p*-value = 0.002), respectively, whereas the mean MCV and RDW increased significantly from 82.57 ± 9.71 fL to 87.96 ± 14.32 fL (*p*-value = 0.001) and 48.16 ± 3.5 fL to 51.28 ± 4.0 fL (*p*-value = 0.00), respectively. However, the RBC count (*p*-value = 0.300) and MCH (*p*-value = 0.284) showed no significant changes (Table 3).

**Table 3:** Changes in red cell parameters of donor blood over a 14-day storage period.

Variable	Baseline	7 days of storage	14 days of storage	*p*-value
Hb (g/dL)	13.00 ± 1.99	12.78 ± 2.26	12.87 ± 2.57	0.009
RBC (×10^12^/L)	4.55 ± 0.62	4.50 ± 0.66	4.58 ± 0.74	0.300
MCV (fL)	82.57 ± 9.71	83.43 ± 10.91	87.96 ± 14.32	0.001
MCH (pg)	27.48 ± 4.36	27.21 ± 4.41	27.28 ± 4.42	0.284
MCHC (g/dL)	33.10 ± 2.22	32.60 ± 1.61	30.90 ± 2.08	0.002
RDW-SD (fL)	48.16 ± 3.5	50.24 ± 4.1	51.28 ± 4.0	0.000

Hb: hemoglobin; RBC: Red blood cell; MCV: mean corpuscular volume; MCH: mean corpuscular hemoglobin; MCHC: mean corpuscular hemoglobin concentration; RDW-SD: red cell distribution width-standard deviation.

### Impact of G6PD deficiency on RBC indices of stored donor blood units

The mean values of the RBC indices (Hb, MCV, MCH and MCHC) of G6PD-deficient and G6PD-non-deficient blood during baseline analysis were slightly lower in full-defect blood compared to non-defect blood. However, the mean RBC count remained the same and the RDW was slightly higher in full-defect blood compared to non-defect blood.

G6PD-deficient samples showed significant decreases in Hb concentration (*p*-value = 0.015) and RBC count (*p*-value = 0.025) and a significant increase in RDW (*p*-value = 0.00) by the 7th day of storage whilst donor blood with normal G6PD enzyme activity maintained stable for Hb concentration (*p*-value = 0.161) and RBC count (*p*-value = 0.997) over this period. Additionally, a significant reduction in MCHC (*p*-value = 0.053) and an increase in RDW (*p*-value = 0.000) occurred in donor blood with normal G6PD activity (Table 4).

**Table 4:** Comparison of red blood cell indices between G6PD-deficient (*n* = 8) and non-deficient donor blood (*n* = 49) after 7 days storage.

Variable	Baseline	7 days of storage	*p*-value
Hb (g/dL)			
G6PD defect	12.61 ± 1.64	11.92 ± 1.88	0.015
G6PD no defect	13.06 ± 2.04	12.92 ± 2.31	0.161
RBC (×10^12^/L)			
G6PD defect	4.56 ± 0.44	4.21 ± 0.44	0.025
G6PD no defect	4.55 ± 0.65	4.55 ± 0.68	0.997
MCV (fL)			
G6PD defect	79.23 ± 12.85	79.80 ± 15.13	0.761
G6PD no defect	83.12 ± 9.15	84.02 ± 10.14	0.238
MCH (pg)			
G6PD defect	25.65 ± 5.71	25.59 ± 5.48	0.923
G6PD no defect	27.78 ± 4.10	27.48 ± 4.22	0.333
MCHC (g/dL)			
G6PD defect	31.88 ± 2.45	32.08 ± 1.44	0.746
G6PD no defect	33.29 ± 2.14	32.69 ± 1.64	0.053
RDW-SD (fL)			
G6PD defect	48.90 ± 50	50.99 ± 5.2	0.00
G6PD no defect	48.03 ± 3.3	50.12 ± 3.9	0.00

Hb: hemoglobin; RBC: Red blood cell; MCV: mean corpuscular volume; MCH: mean corpuscular hemoglobin; MCHC: mean corpuscular hemoglobin concentration; RDW-SD: red cell distribution width-standard deviation.

Again, G6PD-deficient samples showed significant decreases in Hb (*p*-value = 0.03) by the 14th day of storage whilst donor blood with normal G6PD enzyme activity maintained a stable Hb concentration over this period (*p*-value = 0.079). Additionally, a significant reduction in the RBC count (*p*-value = 0.03) occurred in G6PD-deficient blood but not in donor blood with normal G6PD activity. There was a general increase in MCV (*p*-value = 0.034) and RDW (*p*-value = 0.05) which occurred in both G6PD-deficient and G6PD-non-deficient blood by the 14th day of storage (Table 5).

**Table 5:** Comparison of red blood cell indices between G6PD-deficient and non-deficient donor blood after 14 days storage.

Variable	Baseline	7 days of storage	14 days of storage	*p*-value
Hb (g/dL)				
G6PD defect	12.61 ± 1.64	11.92 ± 1.88	11.8 ± 2.12	0.03
G6PD no defect	13.06 ± 2.04	12.92 ± 2.31	13.05 ± 2.62	0.079
RBC (×10^12^/L)				
G6PD defect	4.56 ± 0.44	4.21 ± 0.44	4.24 ± 0.46	0.03
G6PD no defect	4.55 ± 0.65	4.55 ± 0.68	4.63 ± 0.76	0.778
MCV (fL)				
G6PD defect	79.23 ± 12.85	79.80 ± 15.13	83.40 ± 17.70	0.034
G6PD no defect	83.12 ± 9.15	84.02 ± 10.14	88.70 ± 13.77	0.00
MCH (pg)				
G6PD defect	25.65 ± 5.71	25.59 ± 5.48	25.71 ± 5.48	0.968
G6PD no defect	27.78 ± 4.10	27.48 ± 4.22	27.53 ± 4.24	0.195
MCHC (g/dL)				
G6PD defect	31.88 ± 2.45	32.08 ± 1.44	30.68 ± 1.81	0.197
G6PD no defect	33.29 ± 2.14	32.69 ± 1.64	30.94 ± 2.13	0.00
RDW-SD (fL)				
G6PD defect	48.90 ± 5.0	50.99 ± 5.2	52.23 ± 5.5	0.05
G6PD no defect	48.03 ± 3.3	50.12 ± 3.9	51.12 ± 3.8	0.00

Hb: hemoglobin; RBC: Red blood cell; MCV: mean corpuscular volume; MCH: mean corpuscular hemoglobin; MCHC: mean corpuscular hemoglobin concentration; RDW-SD: red cell distribution width-standard deviation.

### Microscopic morphological assessment of G6PD-deficient and non-deficient donor blood after storage

Analysis of blood film comments of 57 donor samples presented with 89.8 % of RBC samples with normocytic normochromic and 10.2 % samples with microcytic hypochromic blood pictures from G6PD-non-deficient donors whereas G6PD-deficient donor samples showed 75 % of samples with normocytic normochromic blood picture, 12.5 % with microcytic hypochromic picture and 12.5 % with anisopoikilocytosis during baseline analysis (Table 6).

**Table 6:** Microscopic morphological variations between G6PD-deficient and G6PD-non-deficient donor units.

Variable	Baseline	7 days of storage	14 days of storage
Film comment	n (%)	n (%)	n (%)
G6PD defect			
normocytic normochromic	6 (75)	6 (75)	6 (75)
microcytic hypochromic	1 (12.5)	1 (12.5)	0 (0)
macrocytic hypochromic	0 (0)	0 (0)	1 (12.5)
anisopoikilocytosis	1 (12.5)	1 (12.5)	1 (12.5)
Total	8 (100)	8 (100)	8 (100)
G6PD no defect			
normocytic normochromic	44 (89.8)	46 (93.9)	42 (85.7)
microcytic hypochromic	5 (10.2)	3 (6.1)	1 (2)
macrocytic hypochromic	0 (0)	0 (0)	6 (12.2)
anisopoikilocytosis	0 (0)	0 (0)	0 (0)
Total	49 (100)	49 (100)	49 (100)

After seven days of storage, 93.9 % of samples from G6PD-non-deficient donors presented with normocytic normochromic and 6.1 % with microcytic hypochromic blood pictures whereas 75 % of samples from G6PD-deficient donors were normocytic normochromic, 12.5 % were microcytic hypochromic and 12.5 % had anisopoikilocytosis (Table 6).

**Figure 2:**
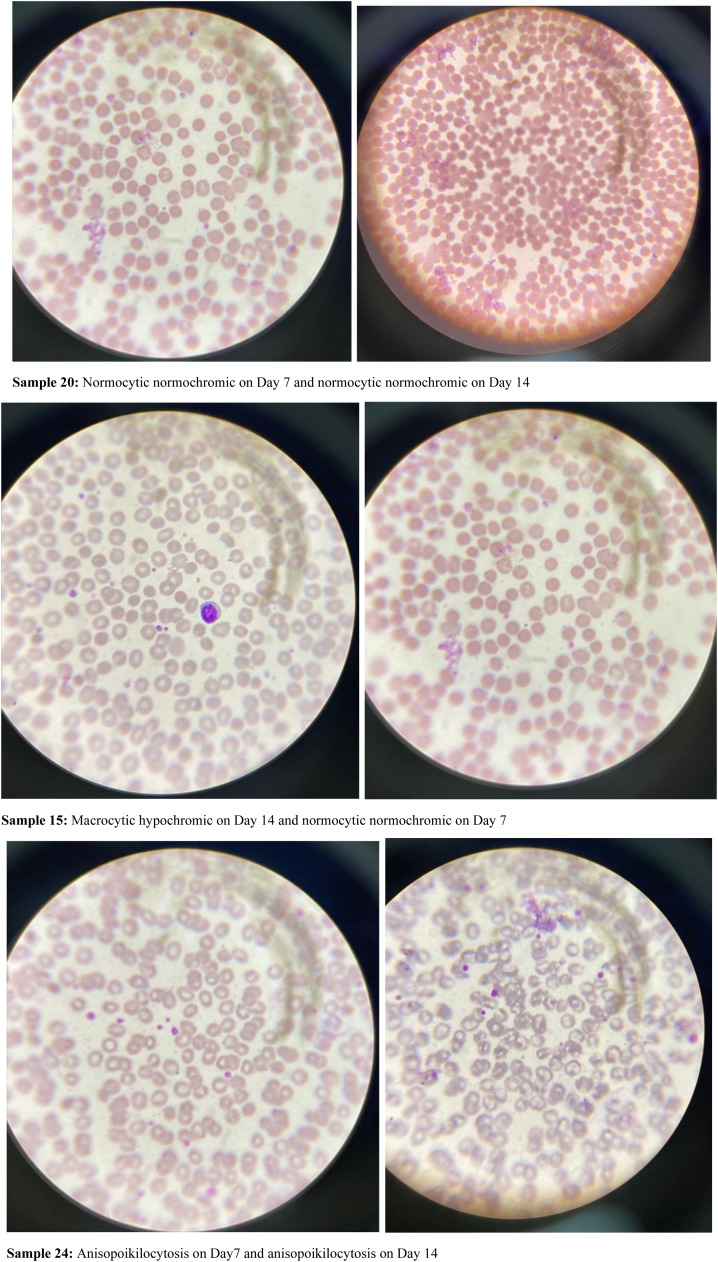
Examples of the film comment results.

Moreover, after 14 days of storage, the blood film comments of G6PD-non-deficient donors identified 85.7 % normocytic normochromic, 2 % microcytic hypochromic and 12.2 % macrocytic hypochromic samples and from G6PD-deficient donor blood 75 % samples were normocytic normochromic, 12.5 % were macrocytic hypochromic and 12.5 % had anisopoikilocytosis (Table 6 and Figure 2).

## Discussion

This study was geared towards establishing the prevalence of G6PD deficiency among blood donors at the Living waters Hospital in the Ashanti region and any potential effect of G6PD enzyme deficiency on RBC indices during storage in the blood bank. The study recruited 57 blood donors all of whom were male with the majority being between 21 and 25 (46.6 %) and 26–30 (36.2 %) years old. The finding on males is that men are the dominant gender in blood donations in line with a study conducted at Sokoto in North Western Nigeria where of a total of blood 14,965 donors from January 2010 to July 2013, 14,871 (99.4 %) were males and only 94 (0.64 %) were female.^18^ Most studies in Africa reported a male dominance in blood donation programs: 61 % in Togo,^19^ 71.2 % in Burkina Faso^20^ and 90 % in Ghana.^21^ In a recent survey in Central, Western, and Eastern Franco-phone African regions, all seven countries surveyed reported <30 % females in their donor populations.^22^ One contributing factor might be that women do not meet donation cut-off values for hemoglobin due to normal menses, menorrhagia, prenatal iron deficiency anemia and postnatal blood loss. From a cultural perspective also, in various African countries it is more likely for males to donate blood given long-standing beliefs that women are not as physically strong as men.^23^ In Western regions, such as Europe, women were found to have higher rates of adverse reactions, primarily vasovagal events, and were also not as likely to meet hemoglobin cut-off requirements for donation.^24^

The age distribution observed in the present study was very similar to those reported by studies in Kenya, East Africa, where 59 % of voluntary donors were <25 years old,^25^ in Burkina Faso, with a reported mean age of 28.9 ± 7.9 years,^26^ and in Rwanda, where >75 % were <30 years old,^23^ highlighting the fact that young people form the backbone of blood donation in these countries.

ABO distribution in this study showed that blood group O Rh positive (45.6 %) was the most predominant among the donors followed by A Rh positive (24.6 %) and B Rh positive (17.5 %). The rarest blood groups were A Rh negative (1.8 %) and O Rh negative (1.8 %). This finding is similar to a study conducted in Cape Coast, Ghana by Patrick Adu et.al., where O-positive was found predominant in 36.59 % and AB-positive was the least common in 6.33 % of the donations. Another study, also in line with this result, reported that the O-positive group was predominant and AB-positive was the least common.^27^ But other studies have reported different results with A-positive being the predominant group followed by O-positive however AB-positive was still the least frequent.^28^

It was observed that, the prevalence of G6PD deficiency among blood donors was 14 % (8/57) which is higher than the 7.9 % reported by Stephen et al. in Cameroon, Central Africa.,^29^ and slightly lower than the 19.5 % reported by Patrick et.al. at Berekum in the Brong Ahafo region of Ghana.^30^ However, Soheir et.al. reported a prevalence of G6PD deficiency of 4.3 % in Egypt, East Africa.^31^ The differences in prevalence between this study and other studies may be attributed to the variations in population studied including genetic factors, screening methods used and the sample size of the population studied.

Storage of whole blood and components is necessary in order to provide support in many accident emergencies, and for obstetric bleeding and post-partum hemorrhage. Provision and storage of blood and blood components is therefore important in the hospital setting.^32^

This study showed a general significant decrease in the Hb concentration and MCHC levels during storage throughout the study period whereas MCV levels had significantly increased by Day 14 suggesting that osmosis of fluid into the RBC increases during storage as the RBC membrane is impaired; this may ultimately lead to RBC hemolysis. This observation confirms the report of Christian Eze et al. that, as storage time increases, hemolysis increases in stored blood.^33^ In line with this assertion, L'Acqua et al. demonstrated that, transfusion of RBCs stored for longer than 4 wk, considerably increased plasma free Hb.^34^ Additionally, a study by Houxiang et al.,^35^ also showed that free Hb and percentage of free to total Hb in storage medium also significantly increased after storage as adenosine triphosphate and 2,3-difosfoglicerato levels were significantly decreased compared to fresh RBCs.

This study also showed that, despite both G6PD-deficient and non-deficient blood donors fulfilled the minimum Hb concentrations for blood donation, G6PD-deficient donors had lower mean Hb concentrations compared to those of donors with normal G6PD enzyme activity. Additionally, over the course of 14 days storage, the Hb concentration and RBC count significantly decreased in G6PD-deficient blood units with a corresponding significant increase in MCV compared to the baseline which differed from insignificant variations observed in Hb, RBC and MCV of donor units with normal G6PD activity. D'Almeida et al. reported decreases in RBC deformability of 34 % following 4 wk of storage,^36^ while Tsai et al. also demonstrated that prolonged storage causes increases in intracellular potassium and free Hb concentrations in the suspending fluid plasma, resulting in a drop in pH leading to decreased fraction of RBCs that survive after being returned to circulation through transfusions.^37^

The significant drop in RBC count and concentration could be due to increased hemolysis as demonstrated by Mattew et al.,^38^ the impact of G6PD status on RBC storage and transfusion outcomes. This could be the result of increased glycolysis, impaired glutathione homeostasis, and increased purine oxidation.

Studies in which RBCs were exclusively stored in a mannitol-containing additive solution (i.e., SAGM, AS-1, or AS-5) showed a significant decrease in G6PD activity during storage.^39^ In contrast, studies of RBCs in other storage solutions, in general, did not suffer this effect.^10^ Consistent with the finding of decreased G6PD activity in some studies, the trend of declining PPP activity upon stimulation is seen during RBC storage.^15^ Therefore, these varied results may be explained by differences in storage conditions or the methods used to assess G6PD function.

Very few studies have been carried out on the effect of G6PD deficiency on peripheral blood film comment. One study conducted by Sutasir et al. on G6PD deficiency shows that routine staining of peripheral smears reveals polychromasia, representing increased RBC production. So-called bite cells caused by the splenic removal of denatured Hb may be seen as can Heinz bodies (denatured Hb) on the peripheral smear in cases of G6PD deficiency.^40^

Contrary to our findings, there were no significant presentations on peripheral blood film of G6PD-deficient donor blood as compared to normal G6PD donor blood throughout the study period. This difference in findings can be attributed to the small sample size of the present study because of the short period given for the study and the short duration of storage of only 14 days. Significant changes were seen by other researchers from 3 wk.

## Limitations

Because this study was conducted in the era of the COVID-19 pandemic, the rates of blood donation at various health centers were drastically reduced hence the small sample size.

Again because of limited resources, extension of unit monitoring beyond 14 days and inclusion of additional parameters such a cellular oxidative stress indices were not possible.

## Recommendations

Based on the findings, the authors recommend;

The need for a multifacility study with a larger sample size to assess a holistic information on the burden of G6PD deficiency, especially in sub-Saharan Africa. This will enhance donor blood quality during transfusions.

A policy should be formulated for G6PD deficiency screening to be included in the screening list for blood donors. This should be observed in all facilities involved in blood donation.

## Conclusion

The most prevalent enzyme deficiency worldwide is G6PD-deficiency. Overall, despite the strong recommendations of the World Health Organization, screening blood donors for G6PD deficiency is not a common practice, and so blood banks and transfusion services have G6PD-deficient RBCs in their inventories. The RBC count and Hb concentration reduce significantly in G6PD-deficient donor blood units in storage with an associated increase in MCV indicating progressive loss of the cellular membrane homeostatic mechanism that could potentially result in further hemolysis during long term storage.

Transfusion of G6PD-deficient blood units may thus not yield optimum transfusion outcomes. This may show up in individuals with higher underlying oxidative stress, such as newborns, people with sickle cell disease, and those using oxidative drugs, as well as lower post-transfusion reactivity of stored G6PD-deficient RBCs and decreased transfusion efficacy in patients.

## Declaration

I hereby declare that this submission is my own work towards the BSc. Degree in Medical Laboratory Technology and that to the best of my knowledge, it contains no material previously published by another person nor material which has been accepted for the award of any other degree of the university, except for references to other people's work, which have been duly acknowledged.

## Ethics approval and consent to participate

Ethical clearance was obtained from the Committee on Human Research Publications and Ethics (CHRPE) of School of Medicine and Dentistry, Kwame Nkrumah University of Science and Technology before the inception of the study. Management of Living Waters Hospital also gave approval for their facility to be used for this study. Consent was sought from blood donors who were assured of the highly confidential nature of this study.

## Consent for publication

Consent for publication was sought from the different authors involved in the development of this work.

## Availability of data and material

Data of this research is available only on request since is a clinical data.

## Funding

The research work was financed solely by the corresponding author.

## Authors contribution

BS is the principal investigator and carried out the model design and the computational framework. AEC designed the model, the computational framework and the analysis of the data and the writing of the article. SO was involved in reagent preparation, laboratory investigations and data analysis. HD helped in the reagent preparation and laboratory investigations. DFA helped in sample collection and storage monitoring. MO assisted in the manuscript development and editing.

## Abbreviations

G6PD: Glucose 6-phosphate dehydrogenase, NADPH: Nicotinamide adenine dinucleotide phosphate, Hb: Hemoglobin, MCV: Mean cell volume, MCH: Mean cell hemoglobin, RBC: Red blood cell, MCHC: Mean cell hemoglobin concentration, PPP: Pentose phosphate pathway

## Conflicts of interest

Not applicable.
